# Antibiotic treatment of rat dams affects bacterial colonization and causes decreased weight gain in pups

**DOI:** 10.1038/s42003-018-0140-5

**Published:** 2018-09-13

**Authors:** Monica Vera-Lise Tulstrup, Henrik Munch Roager, Ida Clement Thaarup, Henrik Lauritz Frandsen, Hanne Frøkiær, Tine Rask Licht, Martin Iain Bahl

**Affiliations:** 1 National Food Institute, Technical University of Denmark, Kemitorvet, DK-2800 Kgs. Lyngby, Denmark; 2 Department of Veterinary and Animal Sciences, Faculty of Health and Medical Sciences, University of Copenhagen, Grønnegårdsvej 15, DK-1870 Frederiksberg C, Denmark; 3 Department of Nutrition, Exercise and Sports, Faculty of Science, University of Copenhagen, Rolighedsvej 26, DK-1958 Frederiksberg C, Denmark

## Abstract

Intergenerational transmission of bacteria during birth initiates the natural successional development of the intestinal microbiota in mammals. This process can be disrupted by antibiotic exposure, potentially affecting early-life microbiota-dependent metabolic programming. In the present study, we specifically investigate the metabolic consequences of exposing neonate Wistar rats to an antibiotic-perturbed low-diversity microbiota from birth until weaning, without exposing the pups directly to antibiotics. Here, we show that pups born from both amoxicillin and vancomycin-treated dams gain less weight than controls. This was concordant with lower feed intake as well as increased colonic expression of the PYY satiety hormone gene at weaning. The weight difference persists into adulthood even though the initial differences in gut microbiota subsided. Our results demonstrate that early-life exposure to an antibiotic-perturbed low-diversity microbiota is sufficient to cause changes in body weight persisting into adulthood.

## Abstract (web summary)

Monica Tulstrup et al. show that antibiotic treatment of female rats during pregnancy leads to altered intestinal colonization in early life and lower body weight in adulthood. They find that this effect correlates with lower feed intake and increased expression of satiety hormone at weaning.

## Introduction

As the number of people in modern societies with obesity continues to rise, increasing attention is given to the commensal intestinal bacterial communities for their role in affecting energy balance^1–4^. Extensive evidence from observational cross-sectional studies in humans, as well as from controlled intervention trials in rodent models, suggests that the intestinal bacterial communities, which have co-evolved with mammalian hosts throughout their evolution, play an important regulatory role for metabolic functions, including weight regulation, fat storage and appetite^5–9^. A seminal study by Liou et al. demonstrates that the conserved shifts in microbiota induced by gastric bypass surgery, including an increase in relative abundance of *Escherichia* and *Akkermansia*, are sufficient to cause weight loss and decreased fat mass in recipient germ-free mice transplanted with this perturbed microbiota^10^. This effect may be due to changes in appetite regulation, which are known in part to be controlled by bacterial metabolites, such as short-chain fatty acids and secondary bile acids, binding to specific G-protein-coupled receptors on enteroendocrine L cells, thereby triggering release of satiety hormones^11,12^. The microbiota has a rapid and pronounced effect on the L cell transcriptome, and germ-free mice have increased levels of circulating GLP-1 which suppresses the intestinal transit rate^13^. The current understanding of long-term metabolic programming is that the very early phases of life are of pivotal importance^4,14^. During the same time period, an orchestrated successional development of the bacterial community takes place^15^. Exposure of the host to microbiota disrupting compounds, such as antibiotics, interferes with this natural process, and can thereby have long-lasting metabolic consequences^16^. Current knowledge does, however, not provide an unambiguous link between early-life antibiotic exposure and overweight in later life^17,18^, probably because the causations at play are influenced by many different factors, including timing of the antibiotic exposure, diet of the offspring^16^ and class of antibiotic^4,19^, and additionally confounded by factors such as exercise, maternal obesity^20^ and infections during pregnancy^21^.

Results from a series of well-controlled rodent intervention trials addressing the effect of antibiotic exposure on host weight gain have been published in recent years. These studies have mostly focused on early-life exposure to antibiotic compounds and reveal that subtherapeutic antibiotic treatment (STAT) of young mice increases adiposity and hormone levels related to metabolism^4^, transient perturbation of the microbiota by STAT in very early life is sufficient to induce sustained effects on body composition related to obesity^16^ and that lifelong STAT induces microbiota changes and enhances the adiposity and insulin resistance associated with high-fat diet^22^. Antibiotic therapy at clinically relevant concentrations during early life has similarly been shown to have long-term effect on metabolic outcomes and weight gain^19^.

Since early-life disruption of the microbiota by antibiotics thus appears to have consequences for future energy balance regulation, it is important to understand the role of intergenerational transfer of microbiota. A recent study has shown that a diet, which has a lowered content of microbiota-accessible carbohydrates (MAC), reversibly alters microbial composition in mice harbouring a human microbiota within a generation. However, if this diet is sustained over several generations, the changes and loss of bacterial species become irreversible unless the missing microbes are administered with the diet^23^.

In the present study, we investigate the consequences of antibiotic-induced microbial perturbation in Wistar rat dams during the peripartum period on appetite regulation and weight gain in the pups. The dams were orally treated with therapeutic doses of either amoxicillin, which is known to be partly absorbed in the small intestine and enter the mammary glands, or with vancomycin, which is not absorbed into circulation following oral administration. Both of these antibiotics, which are used extensively in human therapy, inhibit bacterial cell wall biosynthesis^24^ and have previously been shown to have marked and similar effects on the microbial community structure in Wistar rats^25^. We find that pups born to antibiotic-treated dams have altered intestinal bacterial community composition in early life compared to controls, which gradually recedes into adulthood. Concurrently, we observe decreased weight gain in pups born to antibiotic-treated dams, which display a sustained lower body weight at 14 weeks. At 4 weeks of age, intestinal gene expression of the satiety hormone PYY in antibiotic treatment groups is higher compared to controls, which is consistent with the observation of decreased body weight gain.

## Results

### Peripartum antibiotics causes lower body weight in pups

**Fig. 1:**
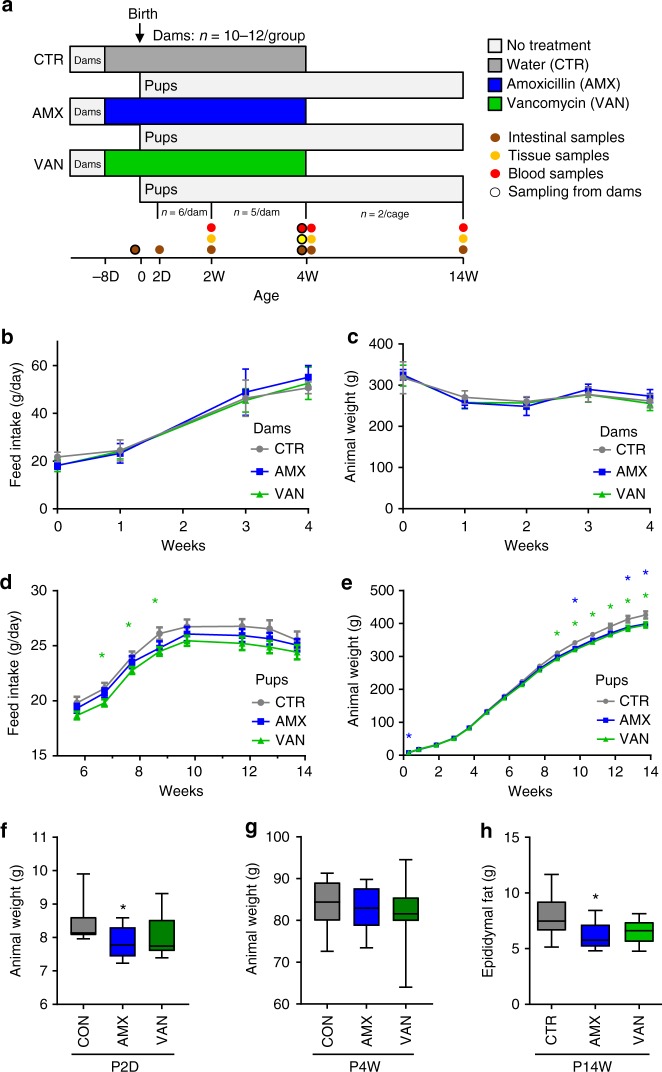
Peripartum antibiotic treatment affects weight gain in pups. **a** Study design. Wistar rat dams (*n* *=* 33) were housed individually and randomized to receive either amoxicillin (AMX), vancomycin (VAN) or water (CTR) by oral gavage daily from 8 days before expected delivery (Day 0) until weaning of their pups at 4 weeks. Pups did not receive antibiotics directly. The litters were reduced to 6 pups per cage (predominantly male) on Day 2 and further at 2 weeks (*n* = 5/cage) and at 4 weeks (*n* = 2/cage) as indicated. Animal weight and feed intake was recorded during the whole study period. Faecal samples were collected from dams just before expected delivery and dissections (intestinal-, tissue- and blood samples) were performed on pups at 4 time points; 2 days (P2D), 2 weeks (P2W), 4 weeks (P4W) and 14 weeks (P14W) as well as the dams at weaning (D4W). Feed intake and animal weight for **b**,**c** dams and **d**,**e** pups was determined weekly and shown as mean ± SEM with *n* *=* 10 (CTR), *n* = 12 (AMX) and *n* = 11 (VAN). Pup weights at **f** P2D and **g** P4W for each treatment group are also shown as box-plots with whiskers indicating total range. **h** Epididymal fat pad weight was determined at termination of pups (P14W) and depicted as box-plots with whiskers indicating total range. Significant differences compared to the CTR group are indicated in all panels: *p < 0.05

In order to investigate the metabolic consequences of exposure to a perturbed microbiota at birth and during early life, we compared groups of rat pups born to dams exposed to either amoxicillin (AMX) or vancomycin (VAN) to untreated controls (CTR) (Fig. 1a). No differences in feed intake or animal weight were observed between groups of dams (Fig. 1b, c). Both AMX and VAN pups appeared to have a slightly lower feed intake from week 6 until termination at week 14; however, the difference was only statistically significant (*p* *<* 0.05, Student’s *t* test) for VAN pups during weeks 7–9 (Fig. 1d). At Day 2, pups (P2D) in the AMX group were found to have significantly lower (*p* *=* 0.025, Student’s *t* test) average body weight than CTR pups (Fig. 1e, f); however, this difference, which could in part be explained by uneven litter sizes at birth until culling at Day 2, disappeared within the first week and no significant differences in weight between groups were observed at weaning of pups at 4 weeks (P4W) (Fig. 1g). From around week 9, the weight of AMX and VAN pups was consistently lower than CTR pups and remained so until termination (Fig. 1e). Furthermore, the epididymal fat pads, a marker of total body fat^26^, were reduced in AMX pups at termination at 14 weeks (P14W) compared to CTR pups and the same trend was observed for VAN pups (Fig. 1h). Administration of antibiotics resulted in significantly increased (*p* = 0.039, Student’s *t* test) spleen weight at termination in AMX dams, but no differences in liver weight (Supplementary Fig. 1). We found no difference in serum levels of the acute-phase protein haptoglobin between groups of pups at week 4 (Supplementary Fig. 2a).

### Antibiotic treatment changes the gut microbiota of dams

**Fig. 2:**
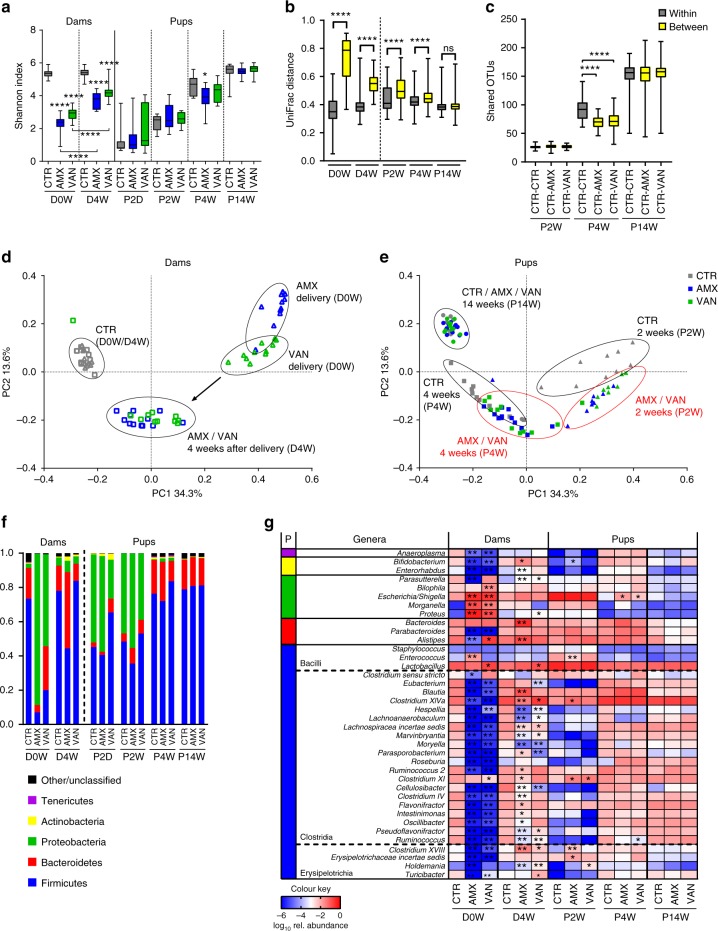
Antibiotic treatment affects microbiota in both treated dams and their pups. **a** Bacterial diversity in faecal/caecal content estimated as Shannon diversity index in dams *n* *=* 10 (CTR), *n* = 12 (AMX) and *n* = 11 (VAN) at the time of giving birth (D0W, faecal sample) and 4 weeks later (D4W, caecal sample) as well as pups at 2 days (P2D, intestinal sample), 2 weeks (P2W), 4 weeks (P4W) and 14 weeks (P14W) (all caecal samples) of age shown as box-plots with whisker indicating total range. **b** Boxplot of unweighted UniFrac distances calculated from caecal microbial composition within all groups (grey) and between groups (yellow) of dams (D0W and D4W) and pups (P2W, P4W and P14W) and **c** boxplot showing numbers of shared OTUs between animals within the CTR group and between the CTR and antibiotic treatment groups of pups at P2D, P4W and P14W. Whisker indicates total range. Principal coordinate analysis (PCoA) based on unweighted UniFrac distances of **d** faecal and caecal samples from dams (D0W and D4W) and **e** caecal samples from pups (P2W, P4W and P14W) coloured according to treatment group. **f** Bacterial composition in dams and pups shown as average relative abundance at the phylum level. **g** Heatmap depicting log_10_ relative abundance scores for faecal and caecal agglomerated genera present in >50% of samples. Results are shown for dams at delivery (D0W) and dams 4 weeks after delivery (D4W) as well as pups at different ages (P2D, P2W, P4W and P14W). In panels **a**, **b** and **c**: *p < 0.05; **p < 0.01; ***p < 0.001; ****p < 0.0001 and in panel **g** FDR-corrected *p* values compared to the CTR group are shown *q < 0.05; **q < 0.01. The left-hand colour bar shows the taxonomic classification at the phylum level

As expected, both amoxicillin and vancomycin dramatically reduced the bacterial α-diversity in the treated dams (Fig. 2a). At the time of delivery (D0W), the faecal microbiota in dams of both treatment groups (AMX and VAN) clustered separately and distantly from the CTR group (Fig. 2b, d) and were generally highly enriched for members of the Proteobacteria phylum, including *Escherichia* spp., *Morganella* spp. and *Proteus* spp., and depleted for Firmicutes, including genera known to produce butyrate, such as *Clostridium XIVa* (Fig. 2f, g). Interestingly, the bacterial diversity in faecal samples from dams, collected ∼1 week after onset of the treatment (D0W), was lower than the diversity in caecal samples taken 4 weeks later (D4W) (Fig. 2a), which suggests a gradual adaptation to the sustained antibiotic pressure, which could in part be driven by antibiotic- resistant strains. In line with this, a principal coordinate analysis (PCoA) based on unweighted UniFrac distances (Fig. 2d), Bray–Curtis dissimilarity (Supplementary Fig. 3a) as well as relative abundance distribution patterns at phylum level (Fig. 2f) showed that the microbiota of the AMX and VAN dams were more similar to the CTR after 4 weeks. We have previously shown that faecal and caecal bacterial diversity and composition in both untreated and antibiotic-treated rats are similar^25^. Notably, the total bacterial load in faecal samples from dams ∼2 days before delivery as determined by culturing was significantly higher (*p* < 0.0001, Student’s *t* test) in AMX and VAN dams than in CTR dams (Supplementary Fig. 4).

### Pups show delayed intestinal microbiota development

For all groups of pups, we observed a gradual increase in bacterial α-diversity reaching approximately the same levels as the untreated dams after 14 weeks (Fig. 2a). The α-diversity was significantly lower (*p* = 0.013, Student’s *t* test) in AMX pups than in CTR pups at 4 weeks, with the same tendency for the VAN pups (Fig. 2a). Analysis of unweighted UniFrac distances within and between groups at different time points as well as PCoA analysis revealed group-specific clustering and significant differences in microbial composition in pups at both 2 weeks of age (P2W) and 4 weeks of age (P4W) (Fig. 2b, e). Separation of treatment groups was significant (*p* < 0.001, Adonis test) at all time points with gradually declining separation (P2W; *R*^2^ = 0.22, P4W; *R*^2^ = 0.14 and P14W; *R*^2^ = 0.088). Analysis based on Bray–Curtis dissimilarity showed similar clustering (Supplementary Fig. 3b). Analysis of the number of shared OTUs between pups within the control group and pups between the control group and antibiotic treatment groups showed that at 4 weeks, but not at 2 and 14 weeks, there were significantly fewer (*p* < 0.0001, Mann–Whitney test) shared OTUs between treatment and the control group, than within the control group (Fig. 2c). This indicated that some of the shared OTUs in the CTR P4W pups are lacking in both the AMX and VAN pups. To further investigate this, we focused on OTUs that were omnipresent in the CTR animals at 4 weeks and determined their prevalence in the antibiotic-exposed pups of the same age (Supplementary Fig. 5a). We noted an interesting pattern for OTU_114 (99% homology to *Alistipes ihumii* strain AP11), which was absent in nearly all animals in both the AMX and VAN groups at P4W groups (whilst present in all CTR animals). We further found that this particular OTU remained absent in all VAN (11/11) and most AMX (9/12) pups to the age of 14 weeks and was also not present in the antibiotic-treated dams D0W at the time of giving birth (Supplementary Fig. 5b). At the genus level, the composition of the microbiota of the 2-week-old pups (P2W) of the antibiotic-treated dams differed significantly from CTR pups in several genera. This included a higher relative abundance of *Clostridium XI*, which contains *Peptoclostridium difficile* (formerly *Clostridium difficile*) in both AMX and VAN pups (*P*_adj_ = 0.019 and *P*_adj_ = 0.022, respectively, permutation-based *t* test) (Fig. 2g). At 4 weeks (P4W), only a few genera differed significantly from the CTR, namely *Escherichia*, which was higher in both AMX and VAN pups (*P*_adj_ = 0.042 and *P*_adj_ = 0.049, respectively), and *Ruminococcus* (*P*_adj_ = 0.049), which was less abundant in VAN pups compared to CTR pups. At the of age 14 weeks (P14W), the pup microbiotas of all groups clustered together (Fig. 2e and Supplementary Fig. 3b) and no differences in UniFrac distances within or between treatment groups (Fig. 2b), nor within or between bacterial groups at genus level were found (Fig. 2g). So the initially reduced diversity in the AMX and VAN pups, indicating a delayed microbial development, was mostly normalized at 14 weeks.

### Peripartum antibiotics affects SCFA production in pups

**Fig. 3:**
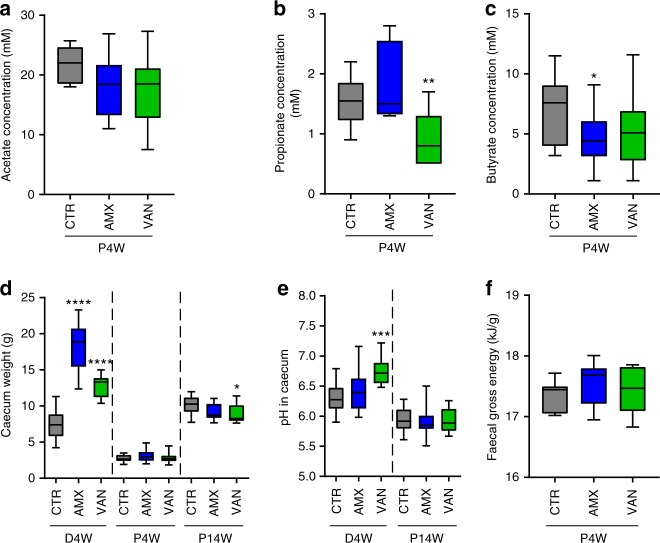
Antibiotic treatment of dams affects short-chain fatty acid levels in pups. Median concentrations of **a** acetate, **b** propionate and **c** butyrate in the caecum of 4-week-old pups. **d** Caecum weight and **e** caecal pH of dams 4 weeks after delivery (D4W) and pups at 4 (P4W) and 14 (P14W) weeks of age. **f** Faecal gross energy in samples from pups at 4 weeks (P4W). All panels show box-plots with whiskers indicating total range. *p < 0.05; **p < 0.01; ***p < 0.001 and ****p < 0.001

To understand whether the observed differences in microbiota in 4-week-old pups caused differences in bacterial activity related to energy extraction and signalling, we investigated short-chain fatty acid composition in the caecum at this age. No significant differences in acetate levels between groups were found (Fig. 3a). Compared to CTR pups, the AMX pups had significantly lower (*p* *=* 0.035, Student’s *t* test) concentrations of butyrate, with the same trend observed for VAN, while the VAN pups also had lower concentrations of propionate (*p* *=* 0.0087, Student’s *t* test) (Fig. 3b, c). The observed reduction in butyrate in the antibiotic groups is consistent with delayed microbial development, but was not accompanied by enlarged caecum size as noted for the dams (Fig. 3d). At 14 weeks of age, the caecum size was found to be slightly reduced in the VAN group compared to the control group; however, this was attributed to a strong correlation between caecum weight and total body weight (Spearman’s rho = 0.62, *p* = 0.0001). The caecal pH of VAN dams was increased compared to CTR dams; however, no difference in pH between the three groups of pups was found (Fig. 3e). Finally, no differences in faecal gross energy were found between the different groups of pups at 4 weeks (Fig. 3f), suggesting that the differences in body weight observed between the groups of pups were not sufficiently explained by differences in microbial energy extraction capacity.

### Appetite regulation is affected by peripartum antibiotics

**Fig. 4:**
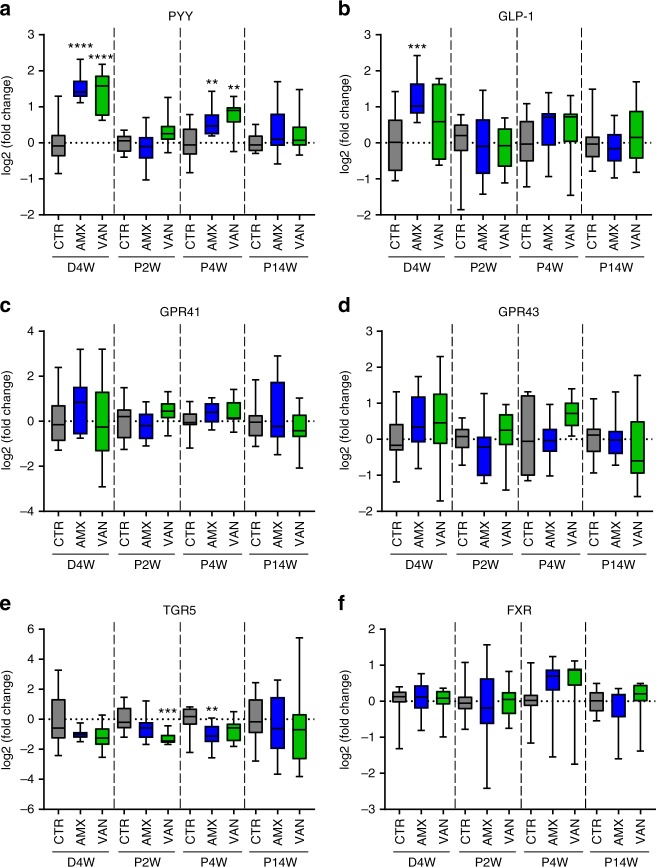
Antibiotic treatment of dams affects gene expression in pups. **a–f** Relative gene expression (compared to the CTR group) in colon tissue from dams 4 weeks after delivery (D4W) and pups at 2 weeks (P2W), 4 weeks (P4W) and 14 weeks (P14W) for appetite hormones (PYY and GLP-1), short-chain fatty acid receptors (GPR41 and GPR43) and bile acid receptors (TGR5 and FXR) determined by quantitative PCR analysis. The results are shown as box-plots with whisker indicating total range of twofold change relative to the average of controls in each age group. In all panels, *p < 0.05; **p < 0.01; ***p < 0.001; ****p < 0.0001

Since we observed a reduced body weight together with a reduced feed intake in the pups that received an antibiotic-perturbed low-diversity microbiota, we considered that expression of intestinal genes potentially affected by the microbiota and related to appetite regulation may be involved. Indeed, expression of the satiety hormone Peptide YY (PYY) gene was higher in both AMX and VAN pups (*p* = 0.0075 and *p* = 0.0012, respectively, Student’s *t* test) at the age of 4 weeks compared to CTR pups, in accordance with the differences observed in the antibiotic-treated dams (Fig. 4a). No differences in PYY expression were observed in pups at 2 weeks despite significant differences in microbial community composition. At the age of 14 weeks also, no differences between groups were observed in the pups, suggesting a transitory effect on colonic PYY gene expression in the pups of antibiotic-treated dams. Colonic expression of the incretin Glucagon-like peptide-1 (GLP-1) gene was also higher in AMX-treated dams (Fig. 4b). However, no difference was observed between groups of pups at any of the sampling times, which was supported by no differences in serum GLP-1 concentrations in 4-week-old pups (Supplementary Fig. 2b). Expression of genes encoding G-protein-coupled receptors GPR41 and GPR43 specific for short-chain fatty acids (SCFAs) did not differ in dams nor pups at any of the sampling points (Fig. 4c, d) despite changed SCFA levels in antibiotic- treated dams and some groups of pups. Expression of the bile acid-specific TGR5 membrane receptor gene in VAN pups was notably lower (*p* = 0.0002, Mann–Whitney test) at 2 weeks, and tended to be lower also at 4 weeks. In AMX pups, the expression of this gene was lower (*p* = 0.0090, Student’s *t* test) at 4 weeks (Fig. 4e). Finally, the expression of the bile acid-sensing nuclear farnesoid X receptor (FXR) was not different between groups of dams and pups, respectively (Fig. 4f). Together, this suggests that changes in colonic gene expression related to appetite regulation may contribute to the observed lower body weight and lower feed intake in the AMX and VAN pups.

### Peripartum antibiotics do not affect bile acid levels in pups

**Fig. 5:**
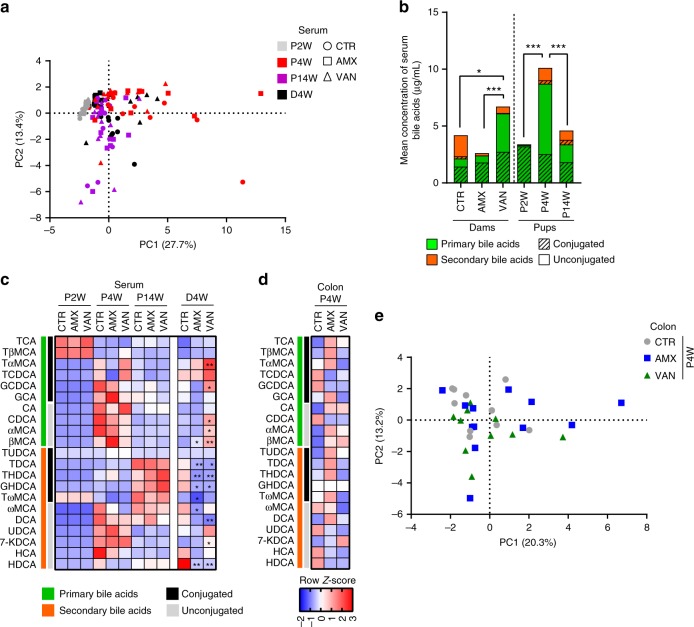
Bile acid concentrations and composition in pups is dependent on age but not peripartum antibiotics. **a** Principal component analysis (PCA) of the serum bile acid profile and **b** total mean concentration of bile acids and distribution of conjugated and unconjugated primary and secondary bile acids, respectively in dams and pups. **c** Temporal and treatment-induced changes in serum bile acid concentrations are visualized in a heatmap based on average row *z*-scores across all groups of samples and shown for pups at 2 weeks (P2W), pups at 4 weeks (P4W), pups at 14 weeks (P14W) and dams 4 weeks after delivery (D4W). **d** Heatmap of the mean abundance (row *z*-score) of the individual bile acids in colon content from pups at 4 weeks. **e** PCA plot of the colon bile acid profiles (P4W). In panel **b** significant differences in total bile acid concentrations are indicated: *p < 0.05; **p < 0.01; ***p < 0.001 and in panel **c**–**d**: FDR-adjusted *p* values compared to the CTR group are shown: *q < 0.05; **q < 0.01. Serum and colonic bile acid concentrations were auto-scaled before PCA to accommodate for highly abundant bile acids and associated loading plots for the PCA plots are shown in supplementary Fig. 6

Since we observed reduced colonic TGR5 expression in pups of antibiotic-treated dams at the age of 4 weeks, we speculated that this could be linked to differences in bile acid profiles resulting from a delayed gut microbiota development in the pups. Antibiotics had a dramatic effect on the serum bile acid profile in dams (Fig. 5a–c), characterized by a reduction of secondary bile acids and an overall increase in bile acids in the vancomycin-treated dams. Principal component analysis (PCA) of serum bile acids in the pups revealed differences according to age, but independent of peripartum antibiotics treatment (Fig. 5a and supplementary Fig. 6a). Serum bile acid profiles were very similar among pups at 2 weeks of age, as shown by the tight clustering of P2W samples in the PCA plot (Fig. 5a). After weaning, at 4 weeks of age, the P4W samples were more scattered (Fig. 5a), probably driven by the microbiota differences observed at this time point (Fig. 2e). Finally, at the age of 14 weeks, the serum bile acid profile of the pups were very similar to that of CTR dams (D4W), as observed by the clustering of P14W samples with the CTR dams (D4W) in the PCA plot (Fig. 5a). The collective mean concentration of serum bile acids in pups differed with age, peaking at the age of 4 weeks, at which time point also secondary bile acids were first markedly observed, and reached a profile similar to that of CTR dams at the age of 14 weeks (Fig. 5b). The bile acid profiles of the 2-week-old pups were dominated by conjugated primary bile acids, while the bile acid profiles at 4 weeks were dominated by de-conjugated primary bile acids, and the bile acid profiles at 14 weeks appeared not to be dominated by any specific bile acid group. The individual serum bile acid concentrations in AMX and VAN pups were not significantly different from CTR pups at any of the time points, possibly due to large inter-individual variations, while several serum bile acids in the antibiotic-treated dams differed from CTR dams (Fig. 5c). In colonic content from pups at 4 weeks, no differences between groups of pups were detected for any of the quantified bile acids (Fig. 5d), although a PCA plot suggested a slightly higher inter-individual variation within the bile acid profile of AMX and VAN pups (Fig. 5e and supplementary Fig. 6b) compared to CTR. Thus, peripartum antibiotics did not significantly affect bile acid profiles in pups.

### Appetite regulation is associated with specific bacterial groups

**Fig. 6:**
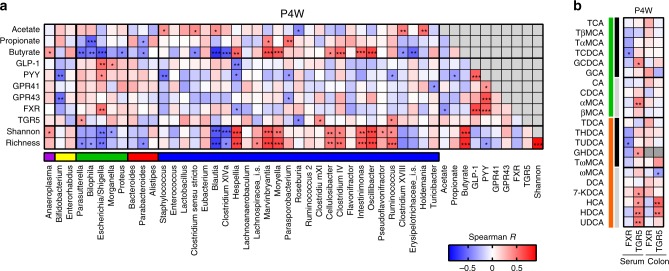
Associations between bacterial genera and intestinal gene expression, short-chain fatty acids and alpha-diversity. **a** Heatmap of Spearman’s rank correlation coefficients between the relative abundance of genera (with a minimum prevalence of 50% across all samples) at 4 weeks of age (P4W) and levels of SCFAs, gene expression and bacterial diversity. The colour bar shows the taxonomic classification of included genera at phylum level (blue: *Firmicutes*, red: *Bacteroidetes*, green: *Proteobacteria*, yellow: *Actinobacteria* and pink: *Tenericutes*). **b** Heatmap of Spearman’s rank correlation coefficients between concentrations of specific bile acids in serum and colon and gene expression in colon of bile acid receptors FXR and TGR5. Colour bars indicate primary (green), secondary (orange), conjugated (black) and unconjugated (grey) bile acids. In both panels, *p < 0.05; **p < 0.01 and ***p < 0.001

To investigate associations between the gut microbiota and intestinal gene expression levels of satiety hormones (GLP-1 and PYY), SCFA receptors (GPR41 and GPR43), bile acid receptors (FXR and TGR5) and caecal SCFA levels in pups at 4 weeks, we conducted a correlation analysis at the genus level (Fig. 6a). Bacterial α-diversity (Shannon and richness indices) in the caecum correlated positively with caecal butyrate levels. Additionally, α-diversity was negatively associated with *Escherichia* spp., which was overrepresented in both antibiotic-treated groups at 4 weeks (Fig. 2g). Notably, *Escherichia* spp. was found to be the only genus that correlated positively with both PYY (Spearman’s rho = 0.41, *p* *=* 0.019) and GLP-1 (Spearman’s rho = 0.52, *p* *=* 0.002) gene expression in colonic tissue and also correlated positively with expression of FXR (Spearman’s rho = 0.50, *p* *=* 0.003). Gene expression of FXR, but not TGR5, was strongly correlated to the gene expression of satiety hormones PYY and GLP-1 (Spearman’s rho = 0.57, *p* *=* 0.0005 and rho = 0.65, *p* *=* 0.00005, respectively). The strongest negative correlations with appetite hormone expression were found for *Staphylococcus* spp. (Spearman’s rho = −0.51, *p* *=* 0.002) and *Hespellia* spp. (Spearman’s rho = −0.47, *p=* 0.006) for PYY and GLP-1, respectively. Colonic expression of bile acid receptors TGR5 and to a lesser extent FXR correlated with several specific primary and secondary bile acids in both serum and colonic content (Fig. 6b). Collectively, only few bacterial groups were found to correlate to expression of appetite hormones GLP-1 and PYY.

## Discussion

Here we report that exposure of conventional Wistar rat dams to either amoxicillin or vancomycin during the peripartum period caused a sustained lower body weight in their male pups, as compared to a control group not exposed to antibiotics. The reduced body weight was also reflected in significantly reduced epididymal fat pats at 14 weeks of age. The pups did not directly receive antibiotics at any time, although a low exposure through coprophagy may occur and additionally amoxicillin may potentially be transferred during lactation due to intestinal absorption in the dams, which is an intrinsic limitation of the experimental design. Previous studies in rodents have, however, indicated such transfer to be minimal^27^. We therefore propose that effects on growth parameters were driven primarily by an antibiotic-induced altered microbial transmission from dams during birth and early life, but cannot rule out direct effects of low doses of antibiotics. This is in line with a recent study demonstrating that colonization of germ-free mice with antibiotic-altered microbial communities may induce changes in the metabolic phenotype^16^. Colonization of germ-free IL10-deficient mouse dams with antibiotic-perturbed bacterial communities as well as peripartum antibiotic treatments of dams has also been shown to enhance colitis in pups^28^. Importantly, these previous studies have indicated a risk of lasting metabolic consequences despite recovery of the intestinal microbial community. In the current study, we found a gradual recovery of the disturbed pup microbiota, which at 14 weeks of age was very similar to the CTR group at the genus level, although grouping was still indicated by the Adonis test based on the UniFrac distance matrix. The number of shared OTUs between animals within the CTR groups at 4 weeks (P4W) was significantly higher than the number of shared OTUs between treatment groups. This observation is a clear indication that some OTUs are absent in the AMX and VAN groups at this time point. An interesting example of this was observed for OTU_114, classified as *Alistipes ihumii*, which is omnipresent in all animals in the CTR group at 4 weeks, but reduced to below-detection level for almost all AMX and VAN pups at P4W and remained so until P14W. In general, *Alistipes* spp. are strict anaerobes and have previously been reported to be underrepresented in the caecum and colon of undernourished mice^29^. At 14 weeks, the body weight of both AMX and VAN pups appeared to approach a plateau significantly lower than that observed for the control group. This observation of stunted growth is different from several recent reports of increased weight gain and adiposity as a consequence of early-life antibiotic exposure^4,22^. However, it is important to recognize the intrinsic variable parameters associated with antibiotic treatment, including the mode-of-action of the antibiotic compound as well as the concentration, timing and duration of exposure. A recent review proposed a pathway for antibiotic-mediated weight modulation inferring that early-life perturbation of the microbial community by high-dose antibiotics can lead to stunted growth due to loss of microbiota-derived calories^30^, which is in line with previous work elucidating the role of the microbiota in relation to energy harvest and fat storage^5,31^. Consistent with our findings, it was recently reported that stunted growth of malnourished Malawian children is associated with delayed maturation of the gut microbiota^32^. They demonstrated that introducing *Ruminococcus gnavus* and *Clostridium symbiosum* to this immature microbiota ameliorated growth and metabolic abnormalities in recipient germ-free mice. Interestingly, we found reduced *Ruminococcus* spp. in the VAN-treated animals at 4 weeks and a negative correlation between this genus and gene expression of the satiety hormone PYY. *Ruminococcus bromii* has also previously been linked to obesity and resistant starch degradation in humans^33,34^. Although several studies thus link antibiotic-induced microbial pertubation to changes in metabolic programming, it has, however, also been shown that antibiotic treatment may elicit microbiome-independent changes in host metabolites^35^. Microbiome-independent effects of antibiotics may therefore also contribute to the observed effects in the current study.

The observed differences in weight gain during the first 14 weeks of life may be caused by differences in feed intake, altered energy extraction from the feed or a combination of these. We generally observed a minor reduction in feed intake for antibiotic pups after weaning, especially for VAN pups, which is consistent with a previous study showing reduced feed intake for adult rats treated orally with amoxicillin^25^. No difference in faecal gross energy at 4 weeks was found, although differences in nitrogen and fat absorption cannot be ruled out. Since there were also no differences in animal weight and only minor differences in microbial composition between groups of pups at 4 weeks, we find it unlikely that microbiota-driven differences in energy extraction were sufficient to cause the observed 6–7% lower body weight of AMX and VAN pups aged 14 weeks.

We thus considered that early-life programming of appetite regulation could be involved in later-life weight regulation. The bacterial diversity, but not the total bacterial load, in the faeces of the antibiotic-treated dams shortly before birth was significantly reduced as compared to control animals, which is consistent with previous studies^25,36^. It has also previously been shown that the vaginal microbiota is altered by antibiotics during pregnancy^37^. Collectively, it is likely that the initial bacterial seeding of the pups of antibiotic-treated dams was very different from CTR dams. Specifically, the pups were initially exposed to a faecal microbiota characterized by low bacterial alpha-diversity with a relatively high abundance of Enterobacteriales, including *Escherichia* spp., *Morganella* spp. and *Proteus* spp. The increased levels of these facultative anaerobes in the dams may be explained by depletion of butyrate-producing Clostridia causing decreased levels of butyrate^25^, which in turn has been reported to fuel aerobic pathogen expansion due to increased epithelial oxygenation^38^. The high relative abundance of Enterobacteriales in the dams at the time of giving birth was, however, not directly reflected in the pups microbiota before the age of 4 weeks, at which point the alpha-diversity was lower compared to CTR pups and the relative abundance of *Escherichia* spp. was higher in both the AMX and VAN pups. Interestingly, the lower alpha-diversity, the increased PYY expression and the reduced weight first became apparent after the pups had started on solid foods. Leptin ingested during lactation may have initially regulated food intake and growth in all pups similarly^39,40^ so that differences in appetite induced by a low-diversity microbiota only became noticeable after weaning.

*Escherichia coli* has previously been associated with body mass index and appetite regulation^41^. The suggested mechanisms behind this association include expression of the ClpB protein, a mimetic of the host α-MSH hormone known to induce satiety^42^, bacterial production of the tryptophan metabolite indole, which modulates GLP-1 release from enteroendocrine L cells^43^ and bacterial lipopolysaccharide binding to TLR4 receptors in the vagal afferents signalling to the brain^44^. In line with this, we identified significant positive correlations between intestinal *Escherichia* spp. relative abundance and colonic gene expression of satiety proteins GLP-1 and PYY in the pups across all treatment groups as well as significantly higher gene expression of PYY in pups of antibiotic-treated dams at 4 weeks. Antibiotics have previously been shown to increase postprandial secretion of PYY in humans^45^. A link between *Escherichia* spp. and weight regulation is consistent with a previous report of weight loss in germ-free mice receiving caecal microbiota from Roux-en-Y gastric bypass-treated mice characterized by increased abundance of *Escherichia*, *Akkermansia* and *Alistipes*^10^.

We have previously reported that antibiotic treatment causes a decrease in the levels of microbially produced SCFAs and increases caecal size and luminal pH^25^. Also in the present study, antibiotic treatment of pregnant dams increased caecal size and pH, consistent with an observed reduction in butyrate-producing Firmicutes. In the 4-week-old pups of antibiotic-treated dams, we found a reduction in caecal butyrate and propionate levels in the AMX and VAN pups, respectively. Very few differences in microbiota at the genus level were seen at this age, but lower levels of the butyrate-producing *Ruminococcus* spp., were detected in the VAN pups. Propionate activates PYY and GLP-1 release from L cells in humans^46,47^, and it has recently been shown that GPR43 deficiency impaired the release of GLP-1 and PYY after administration of propionate^11^. In the current study, no differences in gene expression of SCFA receptors GPR41 and GPR43 were seen between treatment groups at any age in the pups, which suggests that changes in expression patterns of these receptors did not drive the observed metabolic differences in the pups, although differences at the protein level cannot be ruled out.

Antibiotic treatment of dams had a major effect on serum bile acid profiles characterized by a reduction of secondary bile acids in both AMX and VAN groups as compared to CTR. Since the generation of secondary bile acids largely depends on microbial transformation processes, the observed decreased concentration is in accordance with antibiotic-driven alteration of the gut microbiota in the dams and is consistent with previous studies^48–50^. An increase in primary bile acids (both conjugated and de-conjugated) as well as in the total bile acid pool occurred in the VAN dams but not in the AMX dams as compared to the CTR group. The difference between the antibiotic groups is likely to be explained by differences within the lactobacilli, which are resistant to vancomycin and capable of performing de-conjugation via bile salt hydrolase activity^51^.

The differences in bile acid profiles of dams were not transferred to the pups, as bile acid profiles measured in pup serum at ages of 2, 4 and 14 weeks and in colon content at 4 weeks did not show any differences between the treatment groups. We did, however, find a down-regulation of expression of the TGR5 receptor in both 2- and 4-week-old pups of antibiotic-treated dams, which is an important ligand of secondary bile acids. Bile acids binding to the TGR5 receptor on enteroendocrine L cells may stimulate the secretion of satiety hormones PYY and GLP-1^52,53^ and bile acid infusions have been shown to increase plasma PYY in a dose-dependent manner^54^.

The observed temporal development in serum bile acid profiles with age, irrespective of the treatment group, is consistent with the co-occurring successional development of the gut microbiota. The immature gut microbiota in early life is expected to have limited bile acid biotransformation capacity consistent with the observed low levels of secondary and de-conjugated primary bile acids in 2-week-old pups. This was succeeded by a dramatic increase in total serum concentration of bile acids at 4 weeks, driven primarily by secondary and unconjugated primary bile acids. The total amount of bile acids then decreased again in the 14-week-old pups to resemble the level of bile acids found in control dams. An observed, spike in total serum bile acid levels at 4 weeks has previously been recognized in Wistar rats^55^ and could be related to the shift from breast milk to solid diet during weaning. Indeed, Massimi et al. tracked the levels of CYP7A1 mRNA, encoding a rate-limiting enzyme in the bile acid synthesis pathway in Sprague Dawley rats, and found a distinctive peak around day 22, at which point weaning took place^56^. Coincident with weaning, the leptin levels in rats increase^39^ and leptin has been suggested to regulate bile salt metabolism^57^.

In conclusion, we found that peripartum antibiotic treatment of rat dams caused a delay in successional microbiota development and sustained lower adult body weight in their pups. Concurrent with the lower body weight, we found differences in appetite hormone PYY and bile acid receptor TGR5 expression levels in colonic tissue as well as decreased levels of caecal SCFA levels in 4-week-old pups. These results are consistent with recent studies substantiating a microbial impact on early-life metabolic programming and provide new knowledge concerning potential risks associated with antibiotic administration during pregnancy.

## Methods

### Animals and housing

Time-mated specific pathogen-free female Wistar Hannover rats (*n* = 33), with a weight of 200 ± 10 g, were obtained from Taconic Biosciences (Lille Skensved, Denmark). Upon arrival on gestation day (GD) 8, the animals were housed individually under controlled environmental conditions (12-h reverse light/dark cycles, temperature 21.5 ± 0.3 °C, relative humidity 51.3 ± 3.1% and 8–10 air changes per hour). Animals had access to ad libitum water and feed (Altromin 1314 until giving birth and then Altromin 1324, Altromin Spezialfutter GmbH, Germany) throughout the study. Animal experiments were carried out at the DTU National Food Institute (Mørkhøj, Denmark) facilities. Ethical approval for the study was given by the Danish Animal Experiments Inspectorate with authorization number 2012-15-2934-00089, C2. The experiments were overseen by the DTU National Food Institutes in-house Animal Welfare Committee for animal care and use. Animals were monitored twice a day.

### Experimental design

Following a 5-day acclimatization period, dams were equally allocated into three treatment groups (with 10–12 animals in each group) based on weight. Blinding was not implemented during the animal trial due to practical reasons. Antibiotic treatment of dams started on GD 14, which is 8 days before expected birth, and consisted of a daily dosage of 0.5 mL of antibiotic solution containing 60 mg mL^−1^ amoxicillin, Sigma-Aldrich A8523 (AMX, *n* = 12), 8 mg mL^−1^ vancomycin, Sigma-Aldrich 861987 (VAN, *n* = 11) or water (CTR, *n* = 10) by oral gavage. Antibiotic treatment continued until pups were weaned at 4 weeks of age. The scale weight of pups as well as feed and water intake after weaning was determined weekly during the study until termination at week 14 (Fig. 1a). Litters were reduced to 6 pups per dam at 2 days of age (P2D), 5 pups per dam at 2 weeks of age (P2W) and further to 2 animals following weaning at 4 weeks of age (P4W) with a preference for male animals remaining. Small litters were supplemented with excess pups from other dams within the same treatment group at P2D. At P2D, P2W, P4W and P14W, the culled animals from each litter were dissected for further analysis, including collection of blood, intestinal tissue and content from caecum and colon and weighing spleen, liver and caecum as well as determining pH in the latter (Orion^TM^ 3-star benchtop pH meter, Thermo Fisher Scientific). Faecal samples collected from dams 2 days before expected delivery (D0W) as well as samples taken during dissection of dams at weaning (D4W) were also included for analysis.

### Extraction of DNA and amplicon library preparation

Intestinal samples for DNA extraction were stored at −80 °C until analysis. Community DNA was extracted from faecal samples collected from dams just prior to delivery (D0W), from total gut content of 2-day-old female pups (P2D) and from caecal content of dams and male pups (D4W, P2W, P4W and P14W) using the MoBio PowerLyzer^®^ Power Soil^®^ DNA Isolation Kit (MoBio Laboratories, Carlsbad, CA) according to the manufacturer’s recommendations with minor modifications. A maximum of 200-mg samples was used for extraction and the bead beating step was conducted twice at 30 cycles s^−1^ for 5 min with a 10-min break in-between (Retsch MM 300 mixer mill). DNA concentrations were measured fluorometrically with the Qubit dsDNA HS kit (Life Technologies). The bacterial community composition was determined by amplification and sequencing of the V3-region in the 16S rRNA gene using the Ion Torrent PGM platform (Life Technologies) as previously described^58^. Briefly, the V3-region of the 16S rRNA gene was amplified using a universal forward primer (PBU 5′-A-adaptor-TCAG-barcode-CCTACGGGAGGCAGCAG-3′) with a unique 10–12-bp barcode for each bacterial community (IonXpress barcode as suggested by the supplier, Life Technologies) and a universal reverse primer (PBR 5′-trP1-adapter-ATTACCGCGGCTGCTGG-3′). The PCR products were purified using the MAGBIO HigPrep™ PCR-96 well protocol according to the manufacturer’s recommendations, and DNA concentrations were determined with Qubit HS assay. Three amplicon libraries were constructed containing material from P2D/D0W, P2W/P4W and D4W/P14W, respectively, by mixing equal amounts of PCR products from each original community. Sequencing of the three libraries was performed on 318-chips for Ion Torrent sequencing using the Ion OneTouch™200 Template Kit v2 DL.

### Bacterial community composition

Sequence data in FASTQ format was initially processed in CLC Genomic Workbench (version 8.5, Qiagen, Aarhus, Denmark) in order to de-multiplex and remove sequencing primers, retaining reads only if both forward and reverse primers were correctly identified with 100% homology as previously described^58^. Next, operational taxonomic units (OTUs) were picked de novo using UPARSE^59^ and an OTU table was generated. Taxonomical classification of OTUs was assigned using the Ribosomal Database Project multiclassifier version 2.10.1 and the RDP database^60^ with confidence threshold set to 0.5 as recommended for sequences shorter than 250 bp^61^. Further downstream processing was performed in QIIME^62^. A phylogenetic tree was generated (make_phylogeny.py) and rooted to an archaeal species following alignment of all OTUs. The OTU table was filtered to exclude singletons and include only OTUs assigned as bacteria (excluding the Cyanobacteria/Chloroplast group), and present in at least two samples, resulting in a total of 1426 OTUs and a median read depth of 32,906 (range 13,684–124,303). The QIIME workflow core_diversity_analysis.py was used to generate alpha- and beta-diversity indices and relative abundance tables at different taxonomical levels with a rarefied depth of 13,600 reads, including all samples except P2D. To determine UniFrac distances within and between groups of animals, the make_distance_boxplots.py script was used. The script compare_categories.py was used to perform Adonis tests on bacterial community composition. To determine the number of shared OTUs, the generated OUT table was first rarefied to 10,000 reads per sample using single_rarefaction.py and the script shared_phylotypes.py was used. A heatmap was generated at the genus level, including only those genera observed in a minimum of 50% across all samples and coloured according to average log_10_ relative abundances within each group/time point. The samples from 2-day-old pups (P2D) contained only 180 OTUs in reads assigned to bacteria with a median read depth of 8639 (range 1484–42,149) and were analyzed only for phylum-level distribution.

### Gene expression analysis

Approximately 1 cm of colon tissue from dissected animals (D4W, P2W, P4W and P14W), stored in RNAlater^®^ (Life Technologies) at −80 °C, was homogenized in lysis buffer (MagMAX-96 RNA Isolation Kit; Ambion, Austin, TX) with glass beads using a FastPrep instrument (FP120, Bio 101, Thermo Savant; Qbiogene). Total RNA from the homogenized samples was extracted by MagMAX Express (Applied Biosystems) by using the MagMAX-96 RNA Isolation Kit (AM 1830, Ambion) for tissues, following the supplier’s recommendations. Next, cDNA was produced from ∼500 ng of total RNA by using the High-Capacity cDNA Reverse Transcriptase Kit (Applied Biosystems, Foster City, CA, USA) according to the manufacturer's recommendations. The resulting cDNA was used as template for the qPCR reaction. The TaqMan^®^ Fast Universal Master Mix (2×) (4366073, Applied Biosystems, Thermo Fisher Scientific, Waltham, USA) was used together with specific TaqMan primers to assay for GLP-1, PYY and GPR41, and GPR43, FXR and TGR5 (Supplementary Table 1). The cDNA was amplified in duplicate on a StepOnePlus instrument (Applied Biosystems). Gene expression was calculated by the comparative cycle threshold (CT) method. The geometrical mean of ACTB and HPRT1 gene expression was used for normalization of the qPCR results. The expression of target genes was normalized to the geometrical mean of the reference genes by calculating ΔC_T_ = C_T(target)_ −C_T(GEOMEAN reference)._ Fold change in gene expression was calculated as 2^ −ΔΔCT^, where ΔΔC_T =_ ΔC_T(sample)_ −ΔC_T(calibrator)_ and the mean ΔC_T_ of samples from control rats was used as a calibrator. Log2(ΔΔC_T_) was taken to illustrate the fold change of the treatment groups compared to the control group, centred at zero.

### Short-chain fatty acid analysis

Caecum samples from 4-week-old pups (P4W) were analyzed for acetic acid, propionic acid and butyric acid by gas chromatography–mass spectrometry (GC–MS) by MS-Omics, Copenhagen, based on an established method^63^. Briefly, the samples were homogenized in Milli-Q water by ultrasonication, acidified with HCl, supernatants were transferred to GC-vials and internal standards were added. The samples were then analyzed in random order by GC–MS and processed with software based on the PARAFAC2 model. Quantified values are calculated assuming that 1 mg of faeces corresponds to a volume of 1 µL.

### Faecal gross energy analysis

Faecal gross energy from 4-week-old pups (P4W) was assessed using a BOMB calorimeter C6000 isoperibol (IKA, Staufen, Germany) according to the manufacturer’s recommendations. Briefly, the faecal samples were dried at 50 °C for 2 days before burning them with a fixed amount of benzoic acid (one pill, C723, IKA) as an accelerant. Results were calculated in J g^−1^ according to the isoperibol mode of the device.

### Bile acid profiling

LC–MS-grade acetonitrile, methanol and acetic acid were obtained from Sigma-Aldrich (St. Louis, MO, USA). All aqueous solutions were prepared using ultrapore water obtained from a Millipore Milli-Q Gradient A10 system (Millipore, Bedford, MA). Oasis HLB 1 cc cartridges (30 mg mL^−1^) were purchased from Waters (Milford, MA, USA).

Authentic compounds of 12 unconjugated, 9 taurine conjugated and 5 glycine-conjugated bile acids were ordered from either Sigma-Aldrich (Sigma-Aldrich, Schnelldorf, Germany) or Steraloids (Newport, RI, USA). Of these, four bile acids, which were absent in the rats, were used as internal standards (IS), namely glycoursodeoxycholic acid (GUDCA), dehydrocholic acid (DHCA), 23-nordeoxycholic acid (23-NDCA) and glycolithocholic acid (GLCA). The other 22 bile acids, which were to be quantified, included tauro-ω-muricholic acid (TωMCA), tauro-α-muricholic acid (TαMCA), tauro-β-muricholic acid (TβMCA), tauroursodeoxycholic acid (TUDCA), taurohyodeoxycholic acid (THDCA), taurocholic acid (TCA), glycocholic acid (GCA), glycohyodeoxycholic acid (GHDCA), taurochenodeoxycholic acid (TCDCA), taurodeoxycholic acid (TDCA), ω-muricholic acid (ωMCA), α-muricholic acid (αMCA), 7-ketodeoxycholic acid (7-KDCA), glycochenodeoxycholic acid (GCDCA), β-muricholic acid (βMCA), hyocholic acid (HCA), taurolithocholic acid (TLCA), cholic acid (CA), ursodeoxycholic acid (UDCA), hyodeoxycholic acid (HDCA), chenodeoxycholic acid (CDCA) and deoxycholic acid (DCA). Details on the authentic compounds are provided in Supplementary Table 2.

Stock solutions (1 mg mL^−1^) of 26 bile acids were individually prepared from their authentic compounds. Conjugated bile acids were prepared in 50% methanol and unconjugated bile acids were prepared in methanol. An IS solution containing four bile acids, namely GUDCA, DHCA, 23-NDCA and GLCA (1 mg mL^−1^ each), was prepared in 50% methanol. The other 22 bile acids were mixed (BA mix) and further diluted with 50% methanol to give final concentrations of 0.01 µg mL^−1^, 0.1 µg mL^−1^, 1 µg mL^−1^ and 4 µg mL^−1^. Serum samples from dams at termination and pups at week 2, 4 and 14 were prepared in Eppendorf tubes where an aliquot of the IS solution (50 µL, 1 µg mL^−1^) had been dried with nitrogen. To each tube, 40 µL of serum and 100 µL of acetonitrile were mixed, vortexed and left at −20 °C for 30 min to precipitate the proteins. Lumen content of colon (100–150 mg) from pups at week 4 was diluted 1:2 with the IS solution (4 µg mL^−1^), vortexed for 10 s and centrifuged at 16,000 × *g*, 4 °C for 10 min. Subsequently, 150 µL of colonic evacuate was mixed with 350 µL of acetonitrile, vortexed and then left at −20 °C for 20 min to precipitate the proteins. After precipitation of proteins, serum and colon samples were centrifuged at 16,000 × *g*, at 4 °C for 10 min in a microcentrifuge and each supernatant was loaded to a HLB cartridge (each cartridge was preconditioned with 1 mL of 70% acetonitrile prior to use) to deplete phospholipids. After sample loading, the flow-through fraction was collected in a new tube. Subsequently, each cartridge was washed with 300 µL of 70% acetonitrile, and the flow-fractions were pooled and dried with nitrogen. Dried samples were stored at −20 °C until use.

Serum and colon samples were reconstituted in 50 and 600 µL of 50% methanol, respectively. Serum and colon samples were analyzed in random order in two separate runs. We used a modified protocol from our previous study^58^: for each sample, a volume of 2 µL was injected into an ultra-performance liquid chromatography quadrupole time-of-flight mass spectrometry (UPLC-QTOF-MS) system consisting of a Dionex Ultimate 3000 RS liquid chromatograph (Thermo Scientific, CA, USA) coupled to a Bruker maXis time- of-flight mass spectrometer equipped with an electrospray interphase (Bruker Daltonics, Bremen, Germany) operating in negative mode. The analytes were separated on a Poroshell 120 SB-C18 column with a dimension of 2.1 × 100 mm and 2.7-µm particle size (Agilent Technologies, CA, USA). The column was held at 30 °C and the sampler at 4 °C. The UPLC mobile phases consisted of 0.01% acetic acid in water (solution A) and acetonitrile (solution B). The analytes were eluted using the following gradient: Solvent programming started with a linear gradient from 10 to 25% B in 10 min at a flow rate of 0.3 mL min^−1^. The flow rate was then increased to 0.4 mL min^−1^ as the gradient went from 25 to 60% B until 18 min, where the gradient was increased to 98% B until 20 min. The gradient was kept constant at 98% B until 23 min, where the flow rate was decreased to 0.3 mL min^−1^. The gradient was returned to initial conditions at 23.1 min and re-equilibrated until 25 min. Mass spectrometry data were collected in full scan mode at 2 Hz with a scan range of 50–1000 mass/charge (m/z). The following electrospray interphase settings were used: nebulizer pressure 2 bar, drying gas 10 L min^−1^, 200 °C and capillary voltage 4500 V. To improve the measurement accuracy, external and internal calibrations were done using sodium acetate clusters (Sigma-Aldrich, Schnelldorf, Germany) and in addition a lock-mass calibration was applied (hexakis(1H,1H,2H,perfluoroetoxy)phosphazene, Apollo Scientific, Manchester, UK).

Bile acids were detected by extraction of ion chromatograms using the exact mass of the bile acids ± 0.002 Da and quantified by use of standard curves and IS with similar retention times as listed in Table S1. Data were processed using QuantAnalysis version 2.2 (Bruker Daltonics, Bremen, Germany) and calibration curves were established by plotting the peak area ratios of all of the analytes with respect to the IS against the concentrations of the calibration standards. The calibration curves were fitted to quadratic regression.

### Faecal bacterial load

Serial dilutions of fresh faecal samples collected from dams 2 days before expected delivery (D0W) were prepared in peptone saline diluent and plated on Wilkins-Chalgren agar (WCA, Oxoid) and plate count agar (PCA, Oxoid). Plates were incubated at 37 °C for 2 days under anaerobic and aerobic conditions, respectively, before enumeration.

### ELISA

Blood serum levels of haptoglobin and GLP-1 in 4-week-old rat pups (P4W) were determined by use of commercial sandwich ELISA assays (EKR750 and EKR1992, respectively, Nordic BioSite ApS, Sweden) as recommended by the supplier. Serum samples for haptoglobin measurements were diluted 1:500 and for GLP-1 1:2. All samples were at least assayed in duplicates.

### Data handling and statistics

All statistical analysis was conducted in GraphPad Prism 7 (GraphPad Software Inc., La Jolla, CA) unless otherwise stated. Generally, the unpaired *t* test or the non-parametric Mann–Whitney test, when appropriate, was used to determine differences between the control group and treatment groups. Animal weight of the pups was calculated by averaging the individual weights of all pups in the appropriate cage. Average food intake per animal per day was calculated by dividing the total food intake with the number of animals per cage and the number of days between measurements. Statistical analysis of the 16S rRNA gene sequencing data to identify differentially represented bacterial groups was performed by permutation-based *t* tests with 10,000 iterations in R, and correction for multiple testing by the Benjamini–Hochberg false discovery rate method^64^. Differences in total concentration of serum bile acids in pups at the different time points and between the three groups of dams, respectively, were tested by one-way ANOVA with Tukey’s multiple comparison test. For multivariate analysis of bile acids, the data were auto-scaled and imported into LatentiX (version 2.11) (Latent5) for PCA to assess the variation of the data. Spearman’s rank correlation coefficients were determined to investigate associations between the relative abundance of bacterial genera, alpha-diversity, SCFAs and gene expression as well as between bile acids and FXR and TGR5 gene expression.

## Electronic supplementary material

Supplementary Information

## Data Availability

The 16S rRNA gene sequence datasets generated during the current study are deposited in the NCBI Sequence Read Archive with the accession number SRP131746. All other relevant data are available from the authors.
