# Supplementary material for: Antibiotic treatment of rat dams affects bacterial colonization and causes decreased weight gain in pups
Source: Commun Biol. 2018 Sep 13;1:145. doi: 10.1038/s42003-018-0140-5 (PMC6137057; doi:10.1038/s42003-018-0140-5)
Supplement: Supplementary file 1 — Supplementary Information [file 42003_2018_140_MOESM1_ESM.pdf]

Supplementary Fig. 1

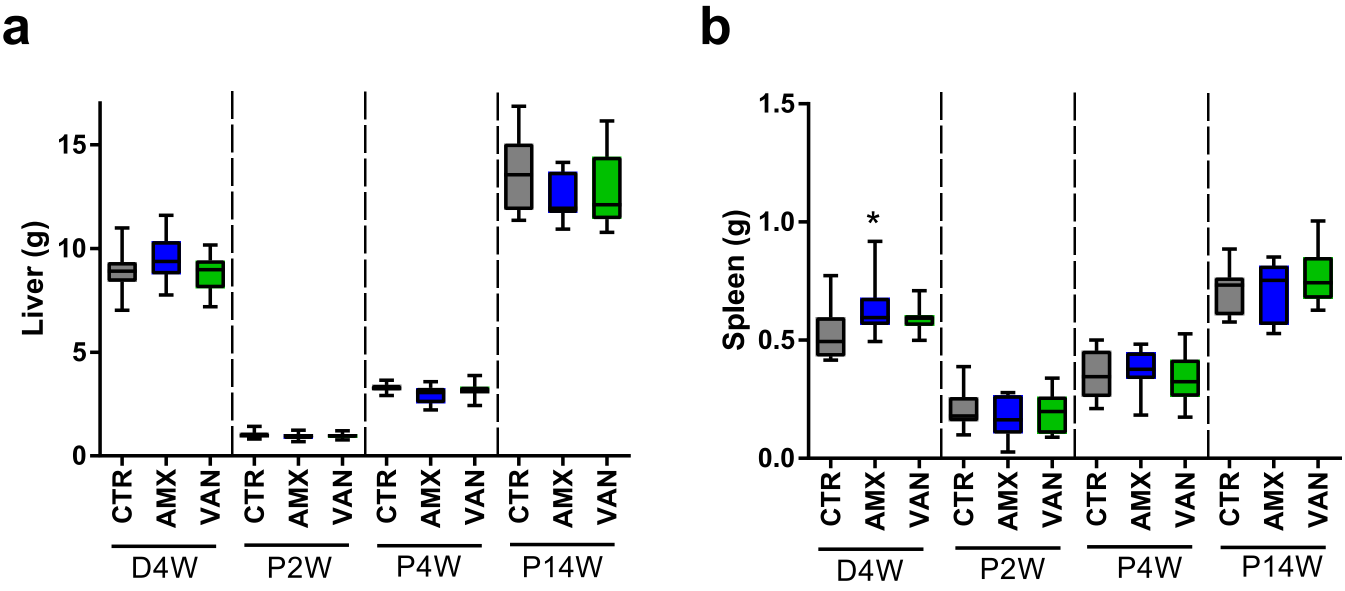

Supplementary Fig. 1 Differences in liver and spleen weight in antibiotic treated dams and their pups. **a** Liver weight and **b** spleen weight of dams 4 weeks after delivery (D4W) and pups at 2 (P2W), 4 (P4W) and 14 (P14W) weeks of age. Panels show box-plots with whiskers indicating total range. In both panels: $^{*}p < 0.05$

Supplementary Fig. 2

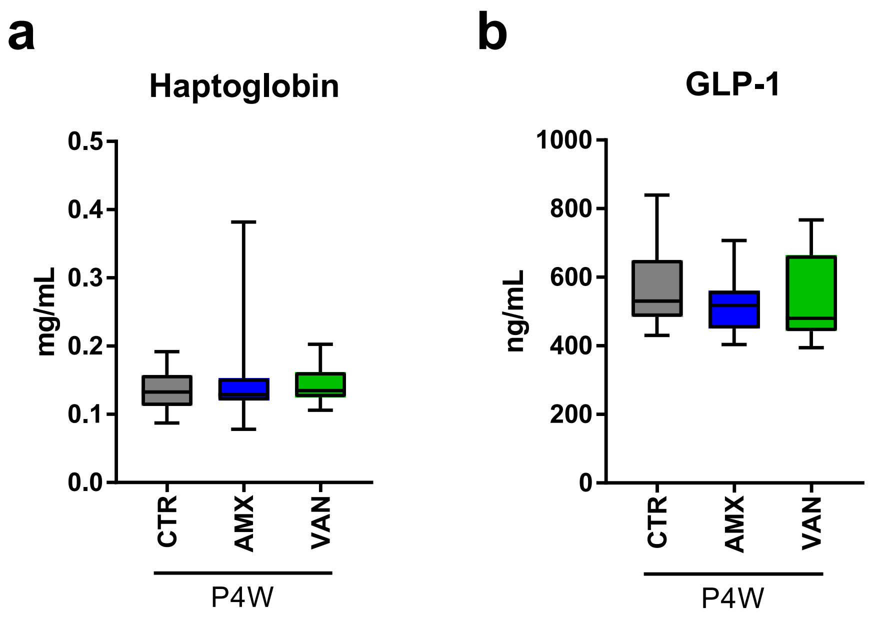

Supplementary Fig. 2 Serum levels of the acute phase protein haptoglobin and GLP-1 in groups of pups at 4 weeks. **a** Haptoglobin and **b** GLP-1 levels are presented as box-plots with whisker showing total range. No significant differences between groups were found.

Supplementary Fig. 3

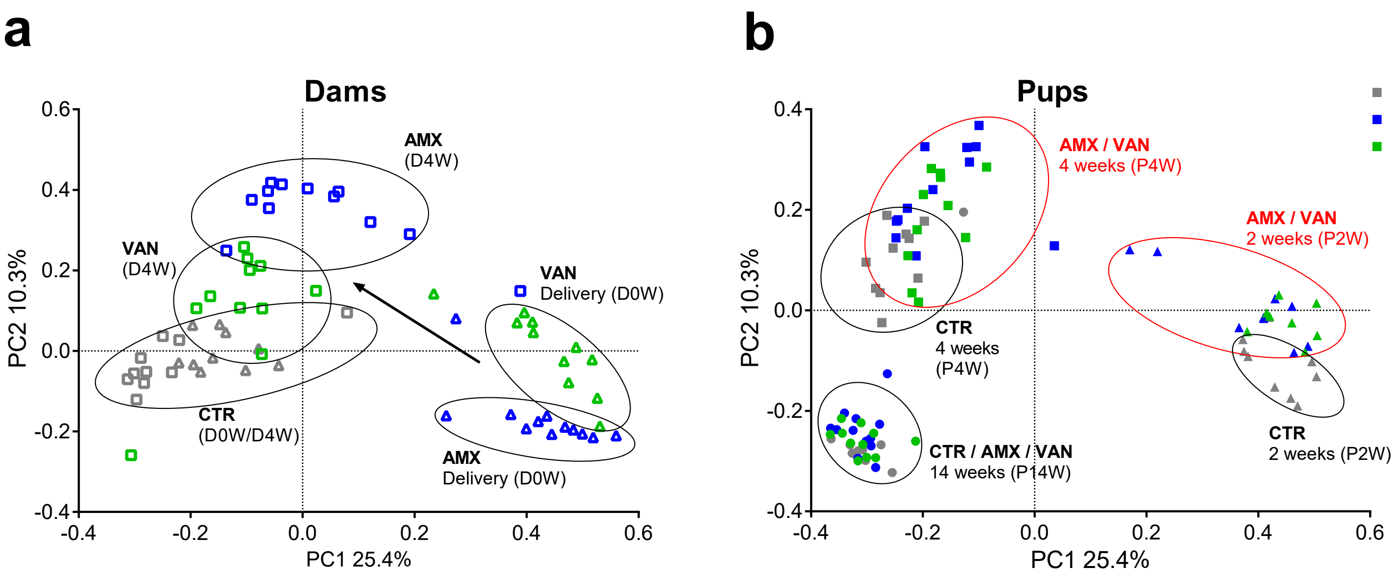

Supplementary Fig. 3 Principle coordinate analysis (PCoA) based on Bray-Curtis dissimilarity of faecal and caecal samples from **a** dams (D0W and D4W) and **b** caecal samples from pups (P2W, P4W and P14W) coloured according to treatment group.

Supplementary Fig. 4

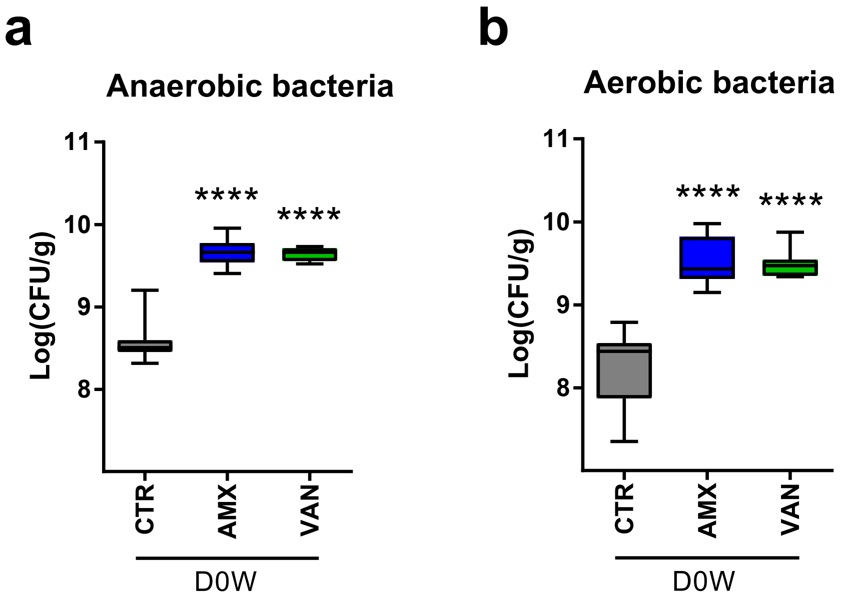

Supplementary Fig. 4 Culturing of faecal samples obtained from dams immediately prior to delivery shows increased total bacterial load in antibiotic treated animal. **a** Total anaerobic bacteria (Wilkins–Chalgren agar) and **b** aerobic bacteria including facultative anaerobic bacteria (Plate-count agar) are presented as box-plots with whisker showing total range. Significant differences compared to the CTR group are indicated in both panels: ****p <0.0001.

Supplementary Fig. 5

a

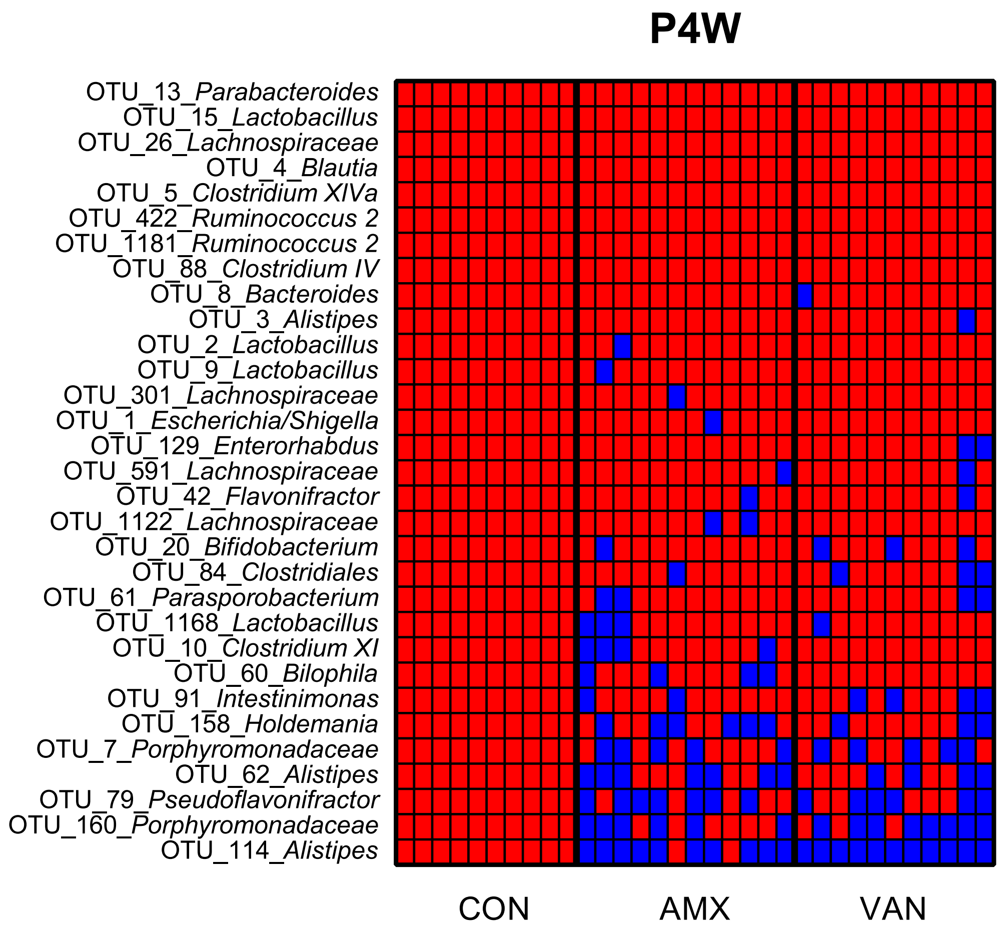

b

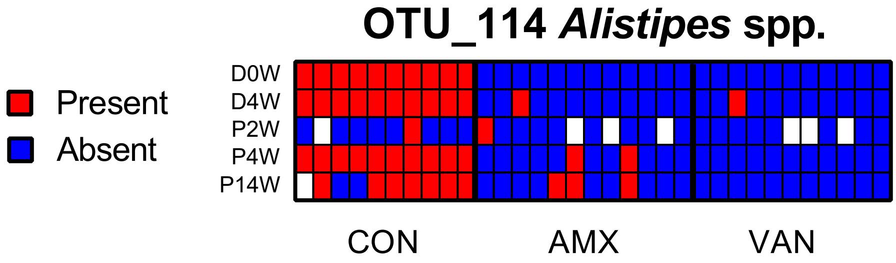

Supplementary Fig. 5 Prevalence of bacterial OTUs in pups of antibiotic treated dams. Heatmaps showing the prevalence of those OTUs found to be omnipresent in CTR pups at P4W in **a** AMX and VAN pups at 4 weeks. **b** Prevalence of OTU_114 classified as *Alistipes* spp. in dams at delivery (D0W) and 4 weeks after delivery (D4W) as well as pups at 2 weeks (P2W), 4 weeks (P4W), and 14 weeks (P14W) for each treatment group.

Supplementary Fig. 6

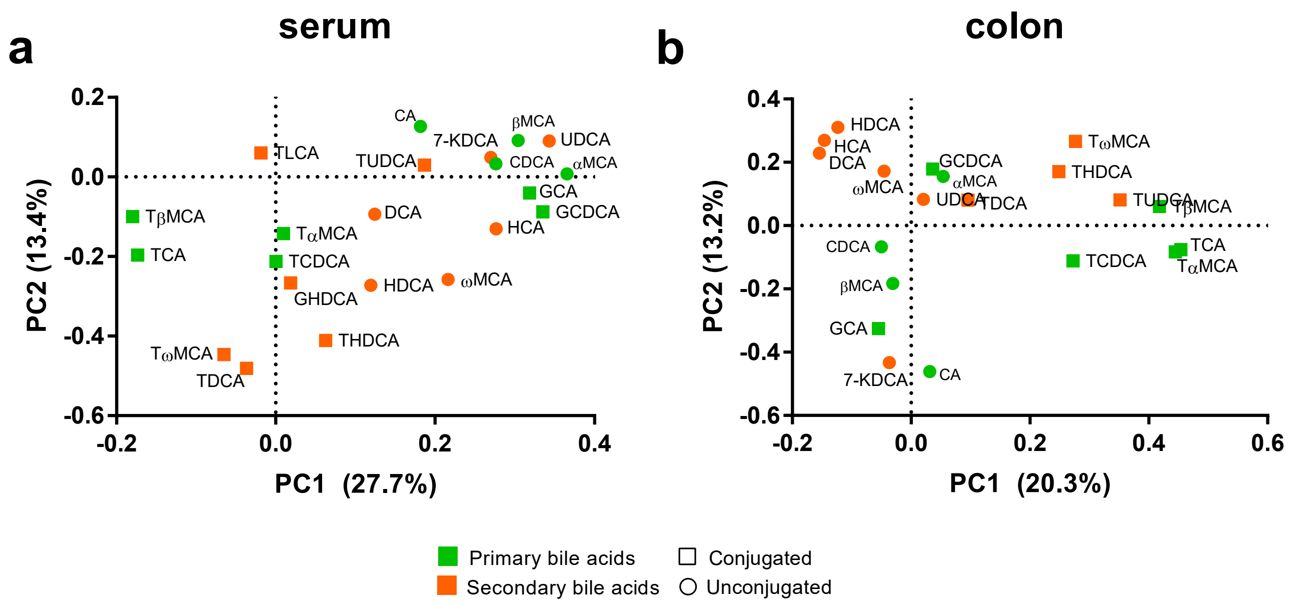

Supplementary Fig. 6 Loading plots for principle component analysis plots of **a** serum bile acid profiles and **b** colon bile acid profiles depicted in Fig. 5.

Supplementary Table 1. List of TaqMan® Assays used in the gene expression analysis

Gene	Name	ID	Probe	Amplicon size<br>*Spans exon
ACTB	actin, beta	Rn00667869_m1	FAM-MGB	91*
HPRT1	hypoxanthine phosphoribosyltrans ferase 1	Rn01527840_m1	FAM-MGB	64*
FXR	nuclear receptor subfamily 1, group H, member 4	Rn00572658_m1	FAM-MGB	95*
TGR5	G protein-coupled bile acid receptor 1	Rn01400316_s1	FAM-MGB	84
GPR41	free fatty acid receptor 3	Rn01457614_g1	FAM-MGB	62*
GPR43	free fatty acid receptor 2	Rn01457833_g1	FAM-MGB	79*
GLP-1	glucagon	Rn00562293_m1	FAM-MGB	65*
PYY	peptide YY	Rn01460420_g1	FAM-MGB	84*

Supplementary Table 2. List of authentic bile acid compounds

No.	Common Name	Abbreviation	Source	Catalogue No.	Molecular Formula	[M-H]-	RT / min	Internal Standard[a]
1	Tauro-ω-muricholic acid	TωMCA	Steraloids	C1889-000	C26H45NO7S	514.2833	10.1	GUDCA
2	Tauro-α-muricholic acid	TαMCA	Steraloids	C1893-000	C26H45NO7S	514.2833	10.4	GUDCA
3	Tauro-β-muricholic acid	TβMCA	Steraloids	C1899-000	C26H45NO7S	514.2833	10.5	GUDCA
4	Tauroursodeoxycholic acid	TUDCA	Steraloids	C1052-000	C26H45NO6S	498.2884	12.0	GUDCA
5	Taurohyodeoxycholic acid	THDCA	Steraloids	C0890-000	C26H45NO6S	498.2884	12.2	GUDCA
6	Taurocholic acid	TCA	Sigma	T4009	C26H45NO72	514.2833	12.2	GUDCA
7	**Glycoursodeoxycholic acid (internal std.)**	**GUDCA**	**Steraloids**	**C1025-000**	**C26H43NO5**	**448.3057**	**12.3**	-
8	Glycocholic acid	GCA	Sigma	G2878	C26H43NO6	464.3007	12.3	GUDCA
9	Glycohyodeoxycholic acid	GHDCA	Steraloids	C0865-000	C26H43NO5	448.3068	12.6	DHCA
10	**Dehydrocholic acid (internal std.)**	**DHCA**	**Sigma**	**30830**	**C24H34O5**	**401.2323**	**13.0**	-
11	Taurochenodeoxycholic acid	TCDCA	Sigma	T6260	C26H45NO6S	498.2884	13.2	DHCA
12	Taurodeoxycholic acid	TDCA	Sigma	T0875	C26H45NO6S	498.2884	13.4	DHCA
13	ω-muricholic acid	ωMCA	Steraloids	C1888-000	C24H40O5	407.2792	13.4	DHCA
14	α-muricholic acid	αMCA	Steraloids	C1890-000	C24H40O5	407.2792	13.6	DHCA
15	7-ketodeoxycholic acid	7-KDCA	Steraloids	C1250-000	C24H38O5	405.2635	13.6	DHCA
16	Glycochenodeoxycholic acid	GCDCA	Sigma	G0759	C26H43NO5	448.3058	13.7	DHCA
17	β-muricholic acid	β-MCA	Steraloids	C1895-000	C24H40O5	407.2792	13.9	23-NDCA
18	Hyocholic acid	HCA	Steraloids	C1850-000	C24H40O5	407.2792	14.3	23-NDCA
19	Taurolithocholic acid	TLCA	Sigma	T7515	C26H45NO5S	482.2935	14.5	23-NDCA
20	Cholic acid	CA	Sigma	C1129	C24H40O5	407.2792	14.8	23-NDCA
21	Ursodeoxycholic	UDCA	Sigma	U5127	C24H40O4	391.2843	15.1	23-NDCA
22	Hyodeoxycholic	HDCA	Sigma	H3878	C24H40O4	391.2843	15.3	23-NDCA
23	**23-nordeoxycholic acid (internal std.)**	**23-NDCA**	**Steraloids**	**N2000-000**	C23H38O4	**377.2686**	**15.6**	-
24	**Glycolithocholic acid (internal std.)**	**GLCA**	**Steraloids**	**C1435-000**	C26H43NO4	**432.3108**	**16.1**	-
25	Chenodeoxycholic acid	CDCA	Sigma	C9377	C24H40O4	391.2843	16.8	GLCA
26	Deoxycholic acid	DCA	Sigma	D2510	C24H40O4	391.2843	16.9	GLCA

Bile acids marked in bold were used as internal standards.

[a]The listed internal standard was used to quantify the given bile acid.
